# Automated classification and explainable AI analysis of lung cancer stages using EfficientNet and gradient-weighted class activation mapping

**DOI:** 10.3389/fmed.2025.1625183

**Published:** 2025-09-10

**Authors:** Abdulmajeed Alqhatani, T. K. S. Rathish Babu, T. R. Mahesh, Surbhi Bhatia Khan, Oumaima Saidani, Mohammad Tabrez Quasim

**Affiliations:** ^1^Department of Information Systems, College of Computer Science and Information Systems, Najran University, Najran, Saudi Arabia; ^2^Department of Computer Science and Engineering, SRM Institute of Science and Technology, Ramapuram, Chennai, India; ^3^Department of Computer Science and Engineering, JAIN (Deemed-to-be University), Bengaluru, India; ^4^School of Science, Engineering and Environment, University of Salford, Salford, United Kingdom; ^5^Division of Research and Development, Lovely Professional University, Phagwara, India; ^6^Department of Information Systems, College of Computer and Information Sciences, Princess Nourah Bint Abdulrahman University, Riyadh, Saudi Arabia; ^7^Department of Computer Science and Artificial Intelligence, College of Computing and Information Technology, University of Bisha, Bisha, Saudi Arabia

**Keywords:** lung cancer staging, EfficientNet, explainable artificial intelligence, Gradient-weighted class activation mapping (Grad-CAM), CT image classification, diagnostic imaging

## Abstract

Precise classification of lung cancer stages based on CT images remains a significant challenge in oncology. This is vitally necessary for determining prognosis and creating practical treatment plans. Traditional methods mainly rely on human interpretation, which can be inconsistent and prone to fluctuation. To overcome these limitations an automated deep learning model based on the EfficientNet-B0 based architecture is proposed. Explainable AI features enhanced through Gradient-weighted Class Activation Mapping (Grad-CAM) help further boost this model. Training of the model was conducted with 1,190 CT scans from the IQ-OTH/NCCD dataset. All the images fell into the benign, malignant, and normal categories. The suggested technique performs remarkably well, reaching 99% accuracy, 99% precision, and recall rates of 96% for benign cases, 99% for malignant cases, and 100% for normal occurrences. Grad-CAM makes the model more interpretable and transparent by providing visual explanations of its results. It identifies the most important regions in the scans that significantly contribute to the classification results. Apart from contributing to the field of medical image analysis, accurate precision and complete explanations also bring automated diagnosis systems credibility and reliability.

## 1 Introduction

Being the major cause of cancer-related mortality worldwide, lung cancer still presents a challenging problem in the field of oncology (1). The stage of the cancer discovery determines much the efficacy of treatment and patient prognosis (1). For best patient treatment, early detection and suitable staging of lung cancer by radiological imaging especially computed tomography are very vital (2). But the way radiologists interpret this imaging data primarily depends on their subjective view, which can vary widely even among experts. This variation might result in unequal and maybe incorrect staging, therefore affecting treatment decisions and results (3).

**Figure 1:**
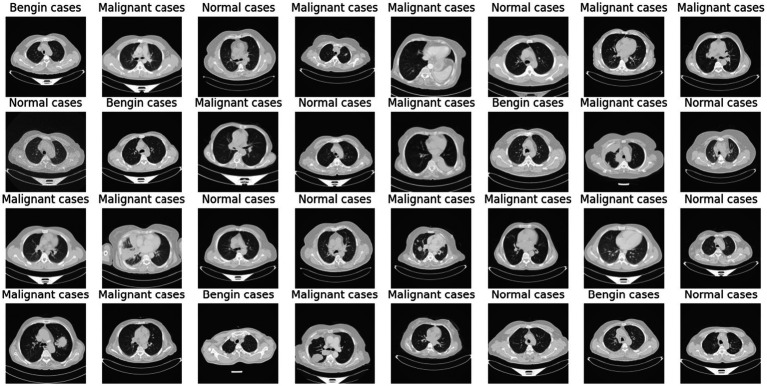
Representative sample of CT images from the IQ-OTH/NCCD dataset.

Deep learning (DL) and artificial intelligence (AI) have opened fresh opportunities to increase diagnosis accuracy in medical imaging (4). With a degree of precision usually approaching human ability, DL models especially convolutional neural networks (CNNs) have shown the ability to identify and comprehend small patterns in picture data (5). Nevertheless, the intrinsic opacity of such models limits their application in clinical practice as the human users of artificial intelligence systems sometimes find their decision-making process unclear or interpretable (6). Figure 1 shows a range of CT scans from the IQ-OTH/NCCD dataset (7, 8), therefore illustrating the range of cases used to train and validate the proposed classification system. The pictures show the range of lung cancer phases: benign, malignant, and normal events. These illustrations provide a graphic summary of the kinds of data used in this work to evaluate the EfficientNet-B0 model and for training.

The major contributions of this research work are as follows:

- The study introduces an automated deep learning model based on the EfficientNet-B0 architecture to classify lung cancer stages (benign, malignant, normal) from CT images, reducing dependence on subjective human interpretation.
- The integration of Gradient-weighted Class Activation Mapping (Grad-CAM) provides clear visual explanations for the model’s decisions, increasing transparency and aiding clinical trust in AI outputs.
- By combining strong classification performance with explainability, the approach supports the development of trustworthy and clinically viable AI systems for automated lung cancer diagnosis.

The objectives of this research can be summarized as follow:

- Construct a deep learning model that can classify lung cancer stages from CT scans with high accuracy, leveraging the EfficientNet-B0 architecture.
- Implement explainable AI techniques, specifically Grad-CAM, to make the model’s decision-making process accessible and interpretable to clinicians.
- Assess the model’s performance in terms of accuracy, precision, and recall, comparing it against existing diagnostic standards to underscore potential improvements and identify any limitations.

This study is organized to first develop a complete overview of the present problems and advancements in lung cancer detection technology. Subsequently, it outlines the suggested methodological approach, utilizing both fresh and proven strategies to solve these issues. The analysis of the data is aimed at confirming the usefulness of the model in real-world scenarios, while the commentary aims to frame this work within the larger context of medical AI research. By boosting both the accuracy and transparency of lung cancer staging, this work contributes to the continuing attempts to incorporate AI into clinical practice, promising considerable gains in patient outcomes through better informed and timely decision-making.

## 2 Literature review

Over the past few years, lung cancer diagnosis has moved significantly from essentially symptom-driven diagnosis to sophisticated imaging-based early detection techniques (1). Because of the late start of symptoms, which severely limited treatment options and significantly affected patient outcomes, lung cancer was historically typically found in later stages (2). Early 2000s low dose computed tomography (LDCT) debut was a breakthrough that allowed a method to detect lung nodules somewhat sooner than conventional radiography (2). By identifying the disease at a more curable stage, studies including the National Lung Screening Trial (NLST) have shown that LDCT screening can reduce lung cancer death (6). Though early identification has improved, the interpretation of imaging data remains a challenge, made worse by high rates of false positives and inter-observer variance in nodule evaluation.

Because CT imaging exactly shows lung anatomy, allowing the identification of tiny lesions not seen on conventional chest X-rays (2), it has become the standard for lung cancer screening and diagnosis. CT scan granularity lets one investigate nodule properties more fully, which is essential for determining cancer risk. Not only in the discovery but also in the stage of lung cancer, the imaging technologies guide biopsy operations and surgical planning. Though they have advantages, the interpretation of CT scans requires great expertise; hence, the little differences between benign and malignant nodules might result in different diagnosis among practitioners.

Table 1 explores the numerous research that are carried out in the area of lung cancer diagnosis and detection and builds a platform for why the research is essential in this field and what were the outcomes of the past research that were carried out in this field.

**Table 1:** Review of recent studies.

Study	Objective	Result	Remarks
Shah et al. (9)	Develop an ensemble 2D CNN approach for detecting lung nodules in CT scans.	Achieved a combined accuracy of 95%.	Utilized the LUNA 16 dataset.
Mikhael et al. (10)	Predict future lung cancer risk from a single LDCT using a deep learning model.	AUC scores ranged from 0.86 to 0.94 across different validation sets.	Model runs in real-time, no additional data required.
Tran et al. (11)	Summarize deep learning applications in lung cancer genomics for decision-making and therapeutics development.	Reviewed various genome-based models.	Focused on omics data and AI integration.
Wankhade and Vigneshwari (12)	Propose a hybrid deep learning method for early lung cancer detection using neural networks.	Confirmed the viability of the hybrid model for early diagnosis.	Used LIDC-IDRI for image extraction.
Wani et al. (13)	Develop an interpretable AI model for lung cancer detection using a hybrid deep learning approach.	Obtained high accuracy and explainability in predictions (accuracy: 97.43%).	Employed the Survey Lung Cancer dataset.
Guan et al. (14)	Create an automated framework for PET image screening, denoising, and segmentation using deep learning.	Demonstrated good performance and time efficiency in tests on real medical PET images.	Focused on lesion tissue segmentation.
Said et al. (15)	Develop a system for early lung cancer diagnosis using deep learning for CT scan image segmentation and classification.	Achieved state-of-the-art performance in segmentation and classification accuracy (97.83 and 98.77%, respectively).	Used the Decathlon dataset for training.
Rajasekar et al. (16)	Analyze features from CT and histopathological images for lung cancer prediction using various deep learning algorithms.	Showed improved performance in detection accuracy compared to existing methods.	Highlighted the significance of combining multiple image types.
Ding et al. (17)	Propose a deep-learning-based method for fast and accurate 3D CT deformable image registration in lung cancer treatment.	Achieved a high average SSIM score and good 3D Gamma passing rates, demonstrating accuracy and efficiency.	Implemented two different models for evaluation.
Zhang et al. (18)	Use deep learning on histopathology images to predict prognosis and therapeutic response in small cell lung cancer.	Developed a pathomics signature with significant prognostic value for survival outcomes and chemoradiotherapy response prediction.	Utilized multicenter cohorts for validation.

Most previous AI lung cancer detection models suffer from overfitting, poor interpretability, and high computational costs, which limit clinical deployment. The model presented here overcomes these shortcomings by combining EfficientNet-B0’s parameter-efficient backbone with strong regularization and Grad-CAM-based explainability. The architecture obtains high accuracy while keeping computation and transparency simple, specifically to fill gaps in previous models that usually utilize large, heavyweight architectures without interpretability.

In contrast to recent directions that promote ensemble and hybrid models for improved accuracy usually at the expense of higher inference time and complexity the presented work takes a lean, single-model architecture approach. EfficientNet-B0’s compound scaling strategy evenly apportions network depth, width, and resolution, producing state-of-the-art performance with less overhead in terms of computation. Grad-CAM also adds confidence by projecting onto areas impacting model predictions, enabling clinical verification.

By combining efficiency, accuracy, and interpretability, this approach presents a practical and scalable solution to real-world clinical lung cancer diagnosis over existing major hindrances of past deep learning methods.

## 3 Methodology

This section describes how to develop and evaluate the deep learning model that is based on CT scans to detect various phases of lung cancer.

**Figure 2:**
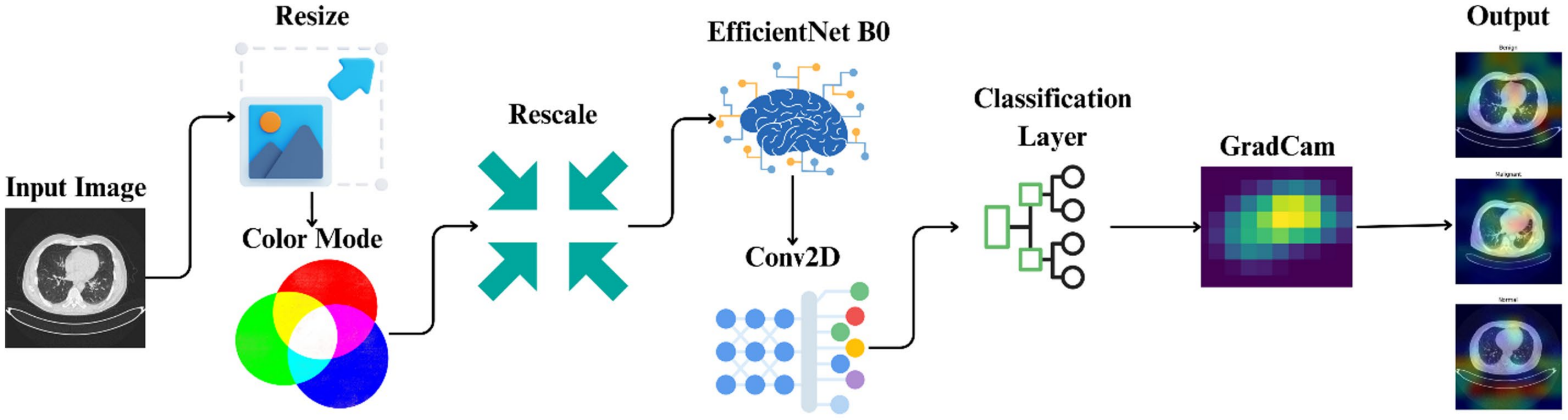
Workflow illustrating the automated classification and explainable AI analysis process for lung cancer staging.

It comprises the utilized dataset, preprocessing methods, model development, training protocols, and the implementation of explainable artificial intelligence technologies. Figure 2 offers a comprehensive overview of methodological methods utilized in this experiment through the illustration of the entire pipeline from image preprocessing to model prediction and explanation. The process begins with preprocessing CT images and proceeds to using EfficientNet-B0 for the training of models. Grad-CAM is then utilized to present visually interpretable explanations of the results of classification.

Algorithm 1 describes the phases and approach for developing a deep learning model to identify lung cancer scans using the EfficientNet-B0 architecture, with specific focus on image preparation, model training, performance evaluation, and interpretability using Grad-CAM visualizations.

**ALGORITHM 1:**
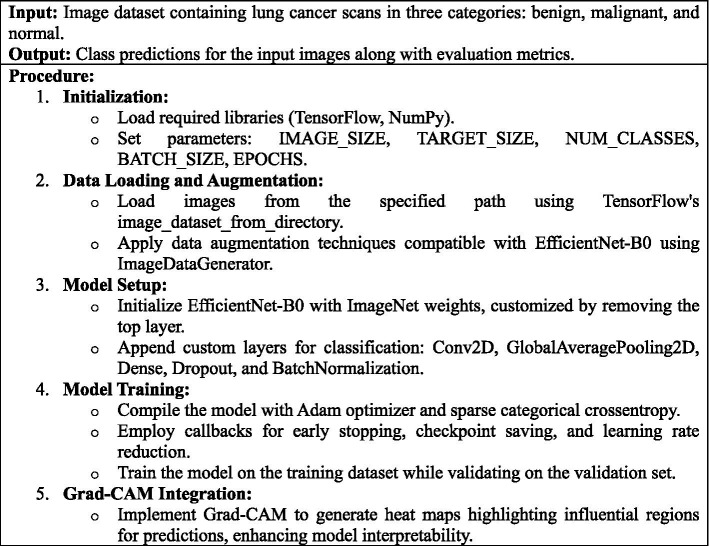
Lung Cancer Classification Using EfficientNet-B0.

### 3.1 Dataset description

The study employed the lung cancer dataset from the Iraq-Oncology Teaching Hospital (IQ-OTH/NCCD) of the National Center for Cancer Diseases (19). This dataset, which is divided into three categories benign, malignant, and normal contains 1,190 CT pictures from 110 people (19). The patients’ demographics included variations in gender, age, and country of origin. The cases were gathered in 2019 over a period of 3 months (19). Every image in the collection shows a different area of the human chest from many views and angles, making them essential for a thorough analysis (19). The dataset component utilized for the training is shown in Table 2.

**Table 2:** Class distribution of dataset.

Class	Number of images
Normal	416
Benign	120
Malignant	561

### 3.2 Preprocessing steps

Preprocessing is necessary to correctly condition the data for interaction with deep learning models. For this study, a rigorous approach was used to ensure that the CT scans from the IQ-OTH/NCCD lung cancer dataset were optimally prepared for processing by EfficientNet-B0 architecture. The initial size of CT scan pictures varies greatly due to their different source. Every picture was downsized to a standard resolution of 224 × 224 pixels to provide a uniform input size for the neural network. This size was selected to strike a compromise between the computational efficiency of processing smaller photos and the requirement to maintain enough image detail. Bilinear interpolation (Equation 1) was used to do the resizing. This technique estimates new pixel values by using the weighted average of the four nearest known pixels, which are positioned diagonally to a given pixel.


(1) $$I(x,y)=(1-a)(1-b)I_{00}+a(1-b)I_{10}+(1-a)bI_{01}+\mathrm{ab}I_{11}$$

Where:

- *I*(*x*,*y*) is the interpolated value at position (*x*,*y*).
- *I*_00_, *I*_10_, *I*_01_, *I*_11_ are the pixel values of the four nearest pixels.
- *a* and *b* are the distances from the pixel (*x*,*y*) to the nearest pixels in the *x* and *y* directions, respectively.

This process aids in the quality preservation of the image during resizing, an important factor in ensuring the integrity of medical images.

The pixel values of the CT images initially range over a large number, characteristic of medical imaging modalities, which can adversely affect the convergence behavior of deep learning models. To overcome this, the pixel values were normalized into 0 to 1 range. This normalization (Equation 2) was done by dividing the pixel values by 255 (the largest possible pixel value in an 8-bit image).


(2) $$p_{\mathrm{norm}}=\frac{p}{P_{\max}}$$

Normalization of the input data was used to alleviate internal covariate shift and hence speed up learning and encourage stable gradient updates during training. The technique minimizes the sensitivity of the model to the different scales of input features, thereby improving overall training efficiency.

Data augmentation methods such as rotation, zoom, and horizontal flip were chosen to mimic natural anatomical variability and symmetry commonly found in clinical CT imaging. These augmentations provide increased data diversity without adding synthetic artifacts that might affect model reliability. Contrast adjustment and noise injection augmentation techniques were specifically not used to avoid maintaining medically important pixel intensities required to accurately interpret clinically.

Even though the initial CT scans were captured in grayscale form, RGB conversion had to be undertaken to meet the pretrained EfficientNet-B0 architecture requirements, which is three-channel input based on its initialization through ImageNet. The conversion, facilitated by duplicating the grayscale channel into all three RGB channels, allows for diagnostic integrity while facilitating successful transfer learning.

All the CT scan images were resized to 224 × 224 pixels to match the input size requirement by EfficientNet-B0. Fixing image size assists with reproducible feature extraction and enables effective batch processing. This resolution was chosen as a compromise between maintaining enough anatomical detail for reliable classification and keeping the computation requirements low. Bilinear interpolation was used for resizing, as it defines each output pixel based on a weighted average of its four closest input pixels, maintaining image smoothness and structural coherence (see Table 3).

**Table 3:** Data split and preprocessing for lung cancer classification.

Parameter	Training set	Validation set	Test set
Data split	60% of original data	20% of original data	20% of original data
Shuffle	Yes	Yes	No
Subset	Training	Validation	N/A

Effective preprocessing such as resizing, normalization, and augmentation not only conditions the data for network input but also improves training stability and model performance. Normalization scales pixel values to a shared range, accelerating convergence and mitigating internal covariate shift. Augmentation adds diversity to the data, preventing overfitting and enhancing generalization. These sequential steps are essential in realizing high diagnostic performance with deep learning models such as EfficientNet-B0.

### 3.3 Model architecture

Proposed deep learning model is developed on top of EfficientNet-B0, which is very well known for its performance and efficiency in handling complex picture data across various fields, including medical imaging. EfficientNet-B0 was chosen as the platform due to its unique architecture, which produces convolutional neural networks (CNNs) more balanced in terms of depth, width, and resolution. This equilibrium avoids the exponential increase in processing costs associated with deeper or larger systems while enabling better performance. Depthwise separable convolutions of Equation 3 and pointwise in Equation 4, which split the convolution operation into two halves that are smaller in size, are important to the EfficientNet-B0 architecture. This approach reduces the computational expense and number of parameters significantly, making the model more efficient without sacrificing its ability to extract useful information from large, complex datasets.


(3) $$\text{Depthwise Convolution}:y[i,j,k]=\sum_{m,n}x[\begin{array}{l} i+m,j+\\ n,k \end{array}]\cdot w[m,n,k]$$

(4) $$\text{Pointwise Convolution}:z[i,j,k]=\sum_{c}y[i,j,c]\cdot w[c,k]$$

where the convolutional kernel (*w*), the intermediate and output feature maps (*y*) and (*z*), respectively, are represented by the variables (*x*, *y*, and *z*).

Every convolutional block consists of batch normalization (Equations 5, 6) layers, which normalize and scale the activations. By guaranteeing a more stable and balanced distribution of non-linear inputs throughout the training process, this normalization aids in preventing internal covariate shift, a prevalent issue in deep network training.


(5) $$\text{Batch Normalization}:\hat{x}=\frac{x-\mu }{\sqrt{\sigma^{2}+\in }}$$

(6) $$\text{Scaled and Shifted}:y=\gamma \hat{x}+\beta$$

where (μ) and $\left(\sigma^{2}\right)$ are the mean and variance of the batch, (∈) is a small constant to avoid division by zero, and (γ) and (β) are learned parameters.

Replacing the usual ReLU, EfficientNet-B0 incorporates the Swish activation function (Equation 7), which has been empirically proved to help in quicker convergence.


(7) Swish(x)=x·σ(x)

where (σ(x)) is the sigmoid function.

The smooth structure of Swish, distinguished by its non-monotonic and dynamic gating mechanism, enables it to sustain activated neurons across the network, therefore facilitating the flow of gradients and lowering the chance of vanishing gradients.

EfficientNet-B0 base model is fine-tuned using pre-trained weights from the ImageNet dataset such that the network can benefit from learned features of a large and diverse set of generic images. Such transfer learning is particularly beneficial for medical imaging tasks in which labeled data may be sparse or expensive to obtain (20).

To customize the network for the objective of classifying lung cancer stages, unique layers are linked to the pre-trained foundation. These contain additional convolutional layers, global average pooling (Equation 8), and dense layers, culminating in a softmax classifier. The convolutional layer extensions are meant to augment feature maps produced by the underlying model, focusing on information vital to medical imaging. The addition of a Conv2D layer atop EfficientNet-B0 allows the network to further adapt high-level features specifically for the lung cancer classification task, capturing domain-specific details absent from generic pretrained features.

Freezing the base EfficientNet layers prevents catastrophic forgetting of general image features and reduces the risk of overfitting given dataset size. While full fine-tuning may boost accuracy on larger or more diverse datasets, this strategy promotes better generalizability on smaller datasets and facilitates efficient training.


(8) $$\text{Global Average Pooling}:z=\frac{1}{H\times W}\sum_{i=1}^{H}\sum_{j=1}^{W}y[i,j]$$

where (H) and (W) are the height and width of the feature map, and (y) is the feature map before pooling.

The Adam optimizer (Equation 9), with its adaptive learning rate feature, steers the learning process of the model by adjusting weights to minimize the loss function suitably. Table 4 provides information regarding model’s configuration and architecture.


(9) $$\text{Adam Update Rule}:\theta_{t+1}=\theta_{t}-\frac{\eta }{\sqrt{v_{t}}+\epsilon }\cdot m_{t}$$

where $\left(\theta_{t}\right)$ represents the parameters, (η) is the learning rate, $\left(m_{t}\right)$ and $\left(v_{t}\right)$ are the first and second moment estimates, and (ϵ) is a small constant.

**Table 4:** Model architecture and configuration for lung cancer classification.

Parameter	Value
IMAGE_SIZE	256
TARGET_SIZE	(224, 224)
NUM_CLASSES	3
BATCH_SIZE	32
EPOCHS	30
Dropout	0.5
Optimizer	Adam
Loss FUNCTION	Sparse Categorical Crossentropy
Metrics	Accuracy

This advanced architecture not only guarantees that the model attains high accuracy in classifying the stages of lung cancer from CT images but also computational efficiency, allowing its application in clinical environments where timely and accurate diagnosis is paramount. Table 5 offers a detailed description of the model architecture, layer types, output shapes, and the number of parameters for each layer.

**Table 5:** Model summary of the lung cancer classification architecture.

Layer (Type)	Output shape	Parameter
EfficientNet-B0 (Functional)	(None, 8, 8, 1,280)	4,049,571
Top_Conv_Layer (Conv2D)	(None, 8, 8, 32)	368,672
global_average_pooling2d_10 (GlobalAveragePooling2D)	(None, 32)	0
dense_20 (Dense)	(None, 128)	4,224
dropout_10 (Dropout)	(None, 128)	0
batch_normalization_9 (BatchNormalization)	(None, 128)	512
dense_21 (Dense)	(None, 3)	387

The added Conv2D layer on top of EfficientNet-B0 enhances the extraction of features specific to lung cancer CT scans, beyond what is captured by the base model trained on natural images. The base layers were frozen during initial training to retain robust general features and prevent overfitting on the relatively small dataset. This strategy supports model generalizability, with plans for fine-tuning if larger datasets become available.

### 3.4 Training procedures

The model’s training schedule was carefully crafted to take use of the Adam optimizer’s advantages. This optimizer is well known for its ability to adaptively modify learning rates according to the first and second moments of the gradients; this feature greatly accelerates the model’s convergence rate and improves its overall training efficiency. This kind of feature is very useful for datasets that require a lot of computing, like those used in medical imaging applications.

For the loss function, Sparse Categorical Cross entropy was employed. This choice is particularly well-suited for multi-class classification scenarios where class labels are provided as integers, allowing for a more memory-efficient handling of label data compared to one-hot encoding.

In the proposed model, Dropout is applied before Batch Normalization as an empirical design choice. While it is more common to apply Batch Normalization first, placing Dropout before BatchNorm can, in some cases, encourage greater regularization by exposing the normalization layer to a wider distribution of activations. It was observed stable performance with this configuration, though both orders are valid and results may be data dependent.

The early stopping patience of five epochs was chosen empirically to balance between adequate learning and prevention of overfitting, as validated by the observed learning curves.

The model’s performance evaluation encompassed a comprehensive suite of metrics, including accuracy (Equation 10), precision (Equation 11), recall (Equation 12), F2 score (Equation 13), Matthews Correlation Coefficient (MCC) (Equation 14), and Cohen’s Kappa.


(10) $$\text{Accuracy}=\frac{\text{Number of Correct Predictions}}{\text{Total Number of Predictions}}$$

(11) $${\text{Precision}}_{i}=\frac{{\mathrm{TP}}_{i}}{{\mathrm{TP}}_{i}+{\mathrm{FP}}_{i}}$$

where:

-
$\left({\mathrm{TP}}_{i}\right)$ is the number of true positives for class (i) ,
-
$\left({\mathrm{FP}}_{i}\right)$ is the number of false positives for class (i).

(12) $${\text{Recall}}_{i}=\frac{{\mathrm{TP}}_{i}}{{\mathrm{TP}}_{i}+{\mathrm{FN}}_{i}}$$

where:

-
$\left({\mathrm{FN}}_{i}\right)$ is the number of false negatives for class (i).

(13) $$F2\;{\text{Score}}_{i}=\frac{5\times {\text{Precision}}_{i}\times {\text{Recall}}_{i}}{4\times {\text{Precision}}_{i}+{\text{Recall}}_{i}}$$

(14) $$\mathrm{MCC}=\frac{\mathrm{TP}\times \mathrm{TN}-\mathrm{FP}\times \mathrm{FN}}{\sqrt{\left(\mathrm{TP}+\mathrm{FP})(\mathrm{TP}+\mathrm{FN})(\mathrm{TN}+\mathrm{FP})(\mathrm{TN}+\mathrm{FN}\right)}}$$

where:

-
(TN) is the number of true negatives.

An Early Stopping callback was used, designed to stop the training should the validation loss not show improvement over five consecutive epochs, therefore preventing overfitting. This approach not only saves computer resources but also keeps the model from learning noise and pointless trends in the training data.

In the training stage, the Model Checkpoint callback was also rather important as it helped to store the model weights at the epoch with best validation accuracy. This assured that, independent of any possible performance drop in next epochs, the best performing model configuration was maintained.

**Figure 3:**
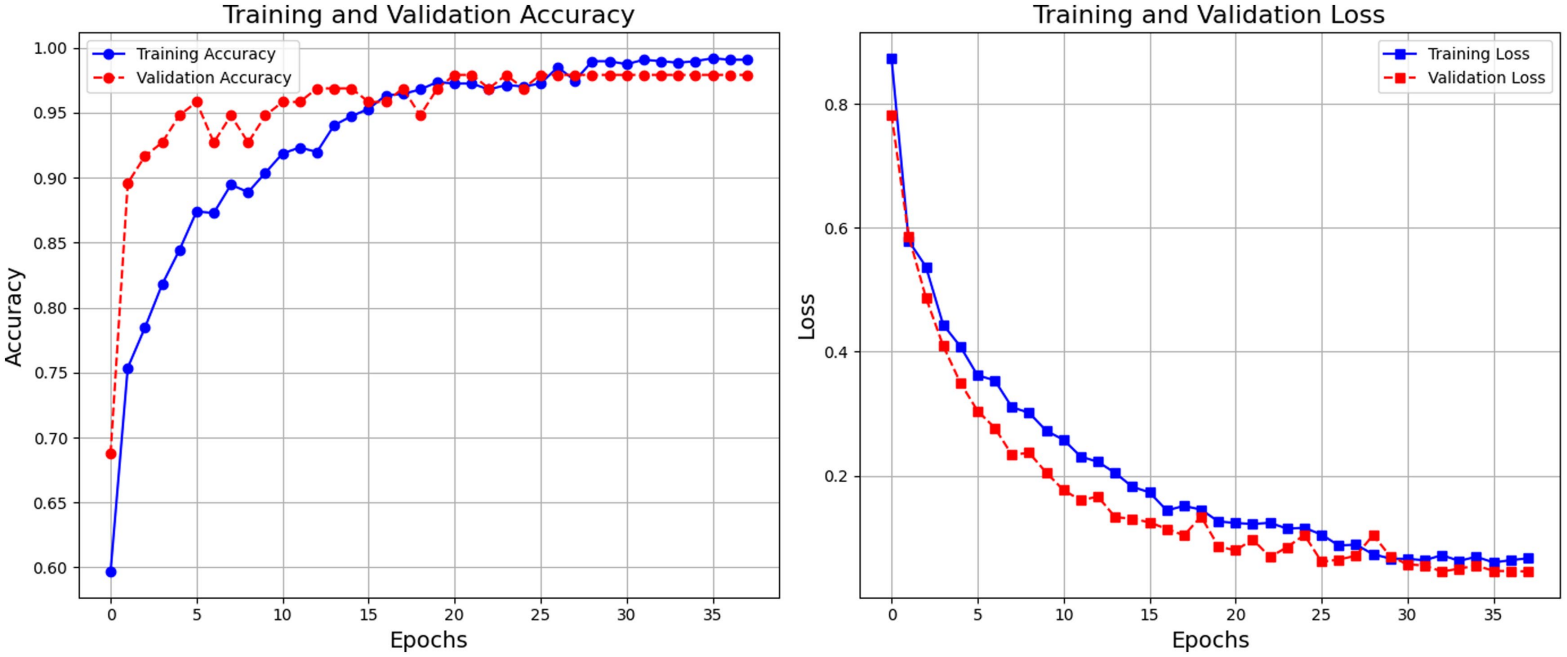
Training and validation accuracy and loss curves across 35 epochs.

Figure 3 depicts the model’s training and validation accuracy curves indicate robust learning with high final accuracy rates. The model exhibits consistent improvement in both training and validation accuracy, stabilizing at around 99% accuracy. The training loss steadily decreases, indicating the model’s learning progression, with minimal overfitting observed.

These strategies, when combined, form a robust framework for training deep learning models, specifically tailored to meet the high standards required in fields like medical imaging, where the accuracy and reliability of predictions can directly impact clinical outcomes.

### 3.5 Integration of explainable AI using gradient-weighted class activation mapping (grad-CAM)

Interpretability is a core expectation for AI-driven diagnostic instruments, especially for medical imaging, since it forms the basis for trustworthiness and clinical validity. To meet this requirement, Grad-CAM is incorporated into CNN models to enhance the transparency of their decision-making. Grad-CAM makes it possible to visualize the parts of an input image that contribute most to the predictions made by a model, thus revealing the features that the model weighs as a priority.

Applying Grad-CAM requires structural adjustments to the CNN. In particular, the model is trained to produce both the final convolutional layer’s activations and the probabilities of the predicted class. This two-output setup is critical for obtaining spatial feature maps and gradient calculations with respect to the target class, which in combination constitute the foundation for creating the Grad-CAM heatmap.

Preserving the outputs of the last convolutional layer retains important spatial information, and the prediction layer determines the target class to be identified. The gradients are calculated by backpropagating the target class score through the network to these spatial features. The gradients provide the contribution of each spatial location to the prediction, which are used as weights highlighting the most significant regions in the input image.

TensorFlow’s Gradient Tape is used as a tool for automatic differentiation to efficiently record these gradients in the forward and backward passes. This process enables accurate and flexible gradient information extraction, promoting solid heatmap generation. The generated heatmaps, overlaid on the input image, offer an easy-to-understand visualization of the areas that inform the decision of the model.

Such visual explanations are especially important in medical imaging. Grad-CAM emphasizes diagnostically meaningful features, including tumors or lesions, so clinicians can check that the decisions of the AI system rely on medically significant regions and not on artifacts or inconsequential areas. This explainability improves clinical validation, helps identify possible model biases, and ultimately encourages trust and deployment of AI-supported diagnostic aids in clinical settings.

## 4 Results

**Figure 4:**
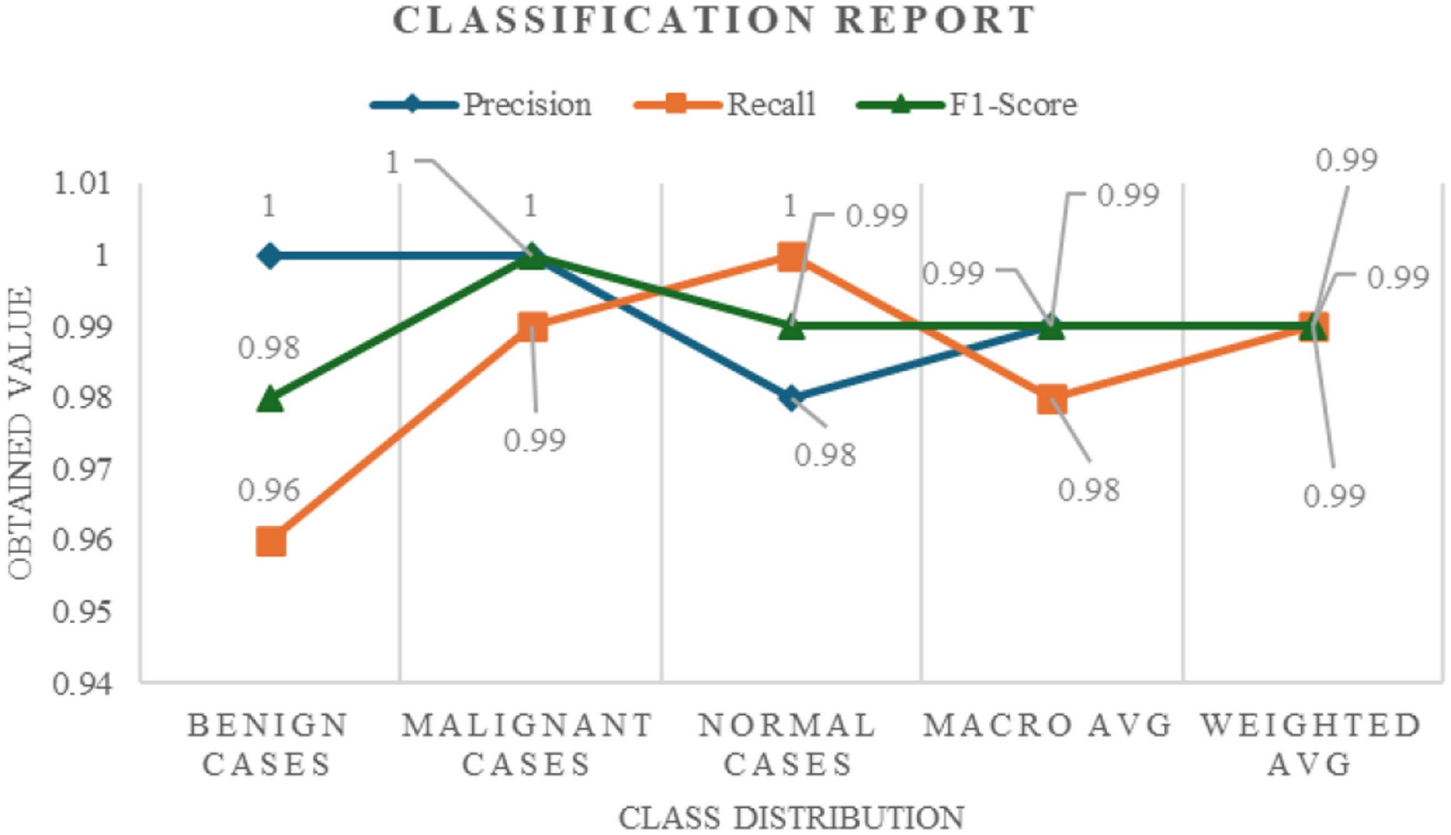
Classification report detailing the precision, recall, and F1-score across different lung cancer stages (benign, malignant, and normal cases).

The performance of the model in classifying lung cancer was evaluated both quantitatively and qualitatively, leading to a comprehensive understanding of its accuracy and reliability. The model had excellent quantitative performance with a Test Accuracy approaching near perfect, which implies perfect classification of all classes in the test dataset. This high level of accuracy was demonstrated by some of the key measures, including recall, precision, F1 and F2 scores, Matthews Correlation Coefficient (MCC), and Cohen’s Kappa. The model demonstrated perfect accuracy for benign, malignant, and normal instances, with 1.00 scores for both classes of instances and 0.98 for normal instances. This accuracy suggests that all the instances correctly predicted to belong to a specific class. All the recall scores of the model, varying between 0.96 to 1.00 for benign, malignant, and normal examples, were quite excellent and proved that it had the ability to identify each instance of each class. The extremely high F1-scores (0.98 for benign, 1.00 for malignant, and 0.99 for normal cases) prove that the model had a good performance on classes and is a balance between accuracy and recall. The model’s bias towards avoiding false negatives, which is essential in medical diagnosis because an omission could have disastrous results, was revealed by the F2 score, which emphasizes recall, and that was 0.9909. Here, highly accurate binary classifications translate very well into multiclass scenarios, as indicated by the MCC score of 0.9845. Figure 4 illustrates the classification report wherein the model’s excellent performance at lung cancer stage classification is manifested through its excellent accuracy, recall, and F1-score over numerous classes.

**Figure 5:**
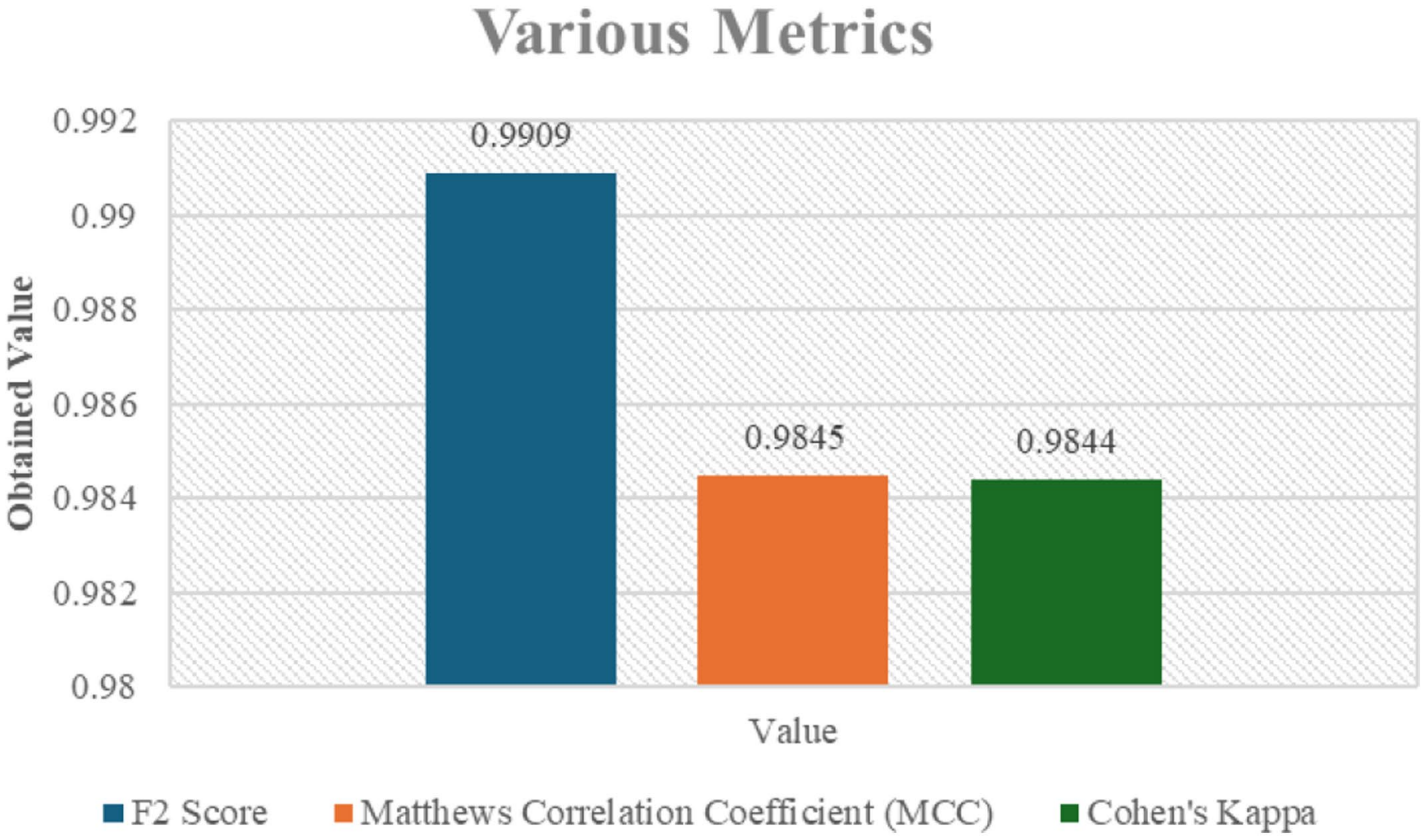
Various metrics including F2 Score, Matthews Correlation Coefficient (MCC), and Cohen’s Kappa for the classification model.

Figure 5 also shows other performance metrics that reaffirm the model’s superior accuracy and reliability in determining lung cancer stages, including the F2 Score, MCC, and Cohen’s Kappa. The resilience of the model is also supported by the F2 value of 0.9909, which indicates superior predictive performance and class balanced accuracy.

**Figure 6:**
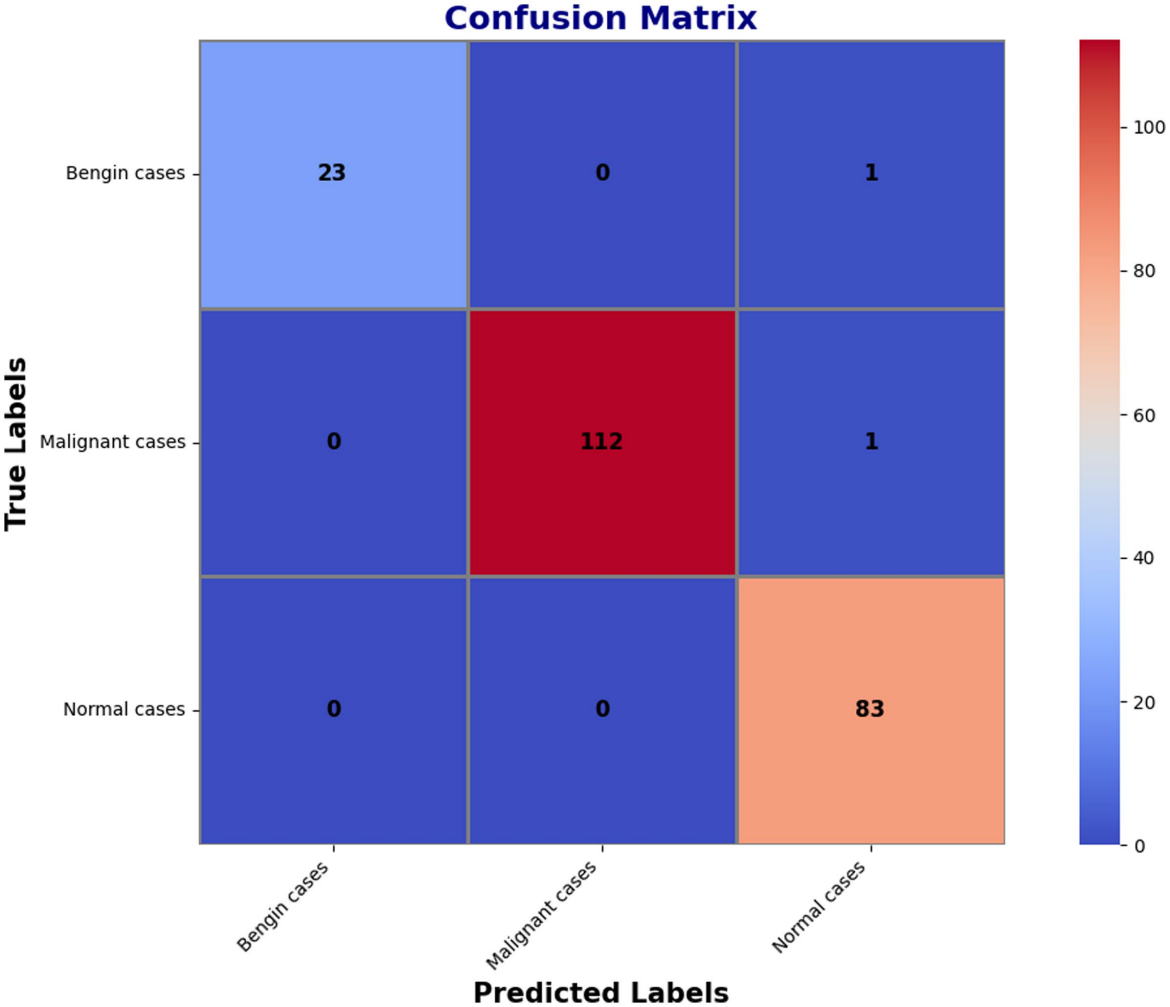
Confusion matrix summarizing the model’s classification performance.

The confusion matrix, which had additional decompositions showing that 23 predictions were correct for benign cases, 112 correct for malignant ones, and 83 correct for normal conditions, further demonstrated the model’s superior dependability. The matrix merely presented a handful of errors: one benign occurrence was misclassified as normal, while one malignant instance was mistakenly identified as normal. These small imperfections reflect how precise the model is when it comes to separating benign, malignant, and normal events. The findings in classification are highlighted in detail within Figure 6’s confusion matrix, which further reflects the high recall and overall accuracy of the model. The matrix confirms that the model’s accuracy is extremely high and the misclassification rate very low when distinguishing between benign, malignant, and normal events.

**Figure 7:**
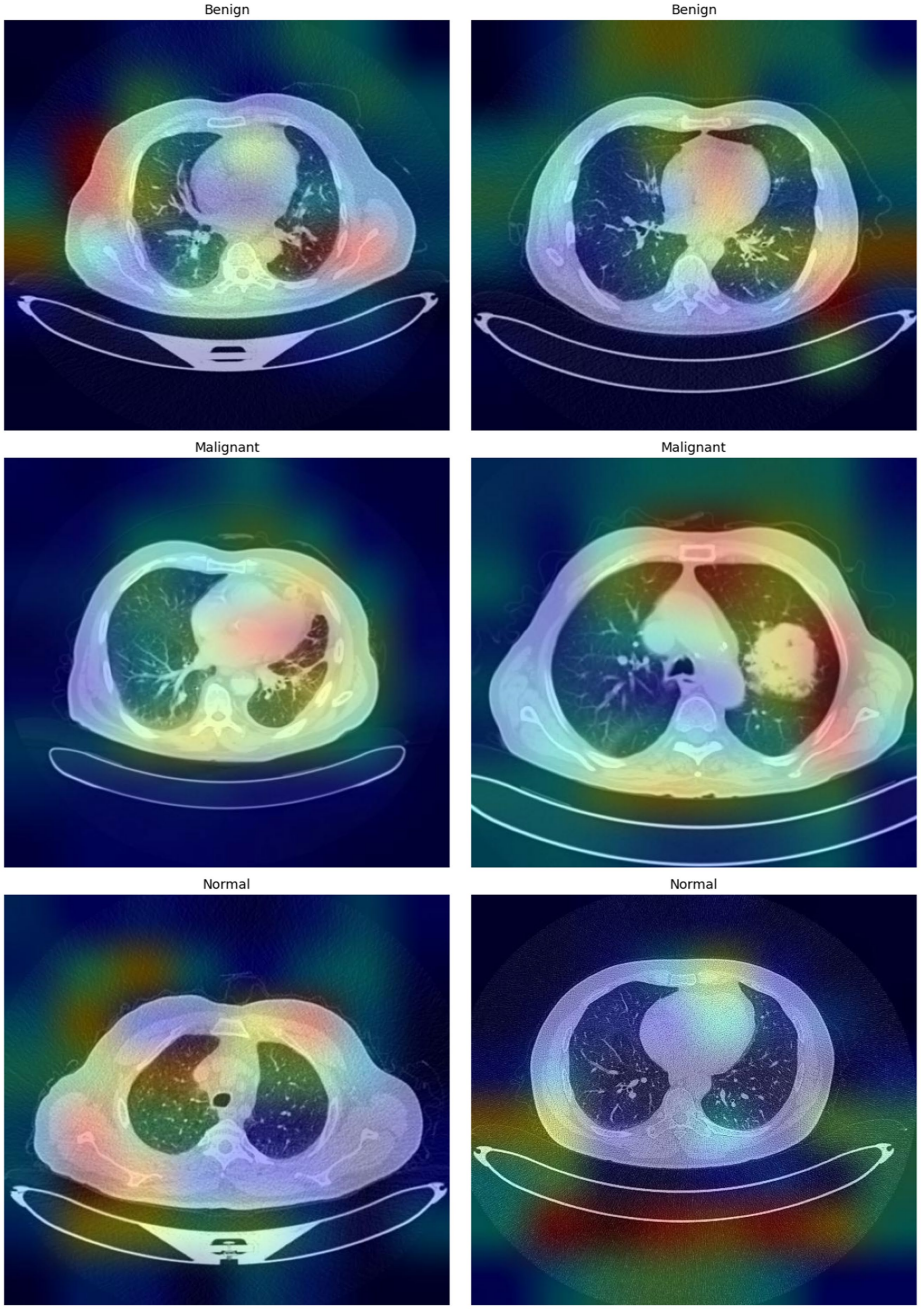
Grad-CAM visualizations highlighting the regions within the CT images that most influence the model’s predictions.

Figure 7 shows the important areas leading the classification decisions, thereby highlighting the interpretability of the model using Grad-CAM images. These heatmaps increase the interpretability and dependability of the automated classification by revealing the areas the model regards as essential for decision-making.

## 5 Discussion

**Figure 8:**
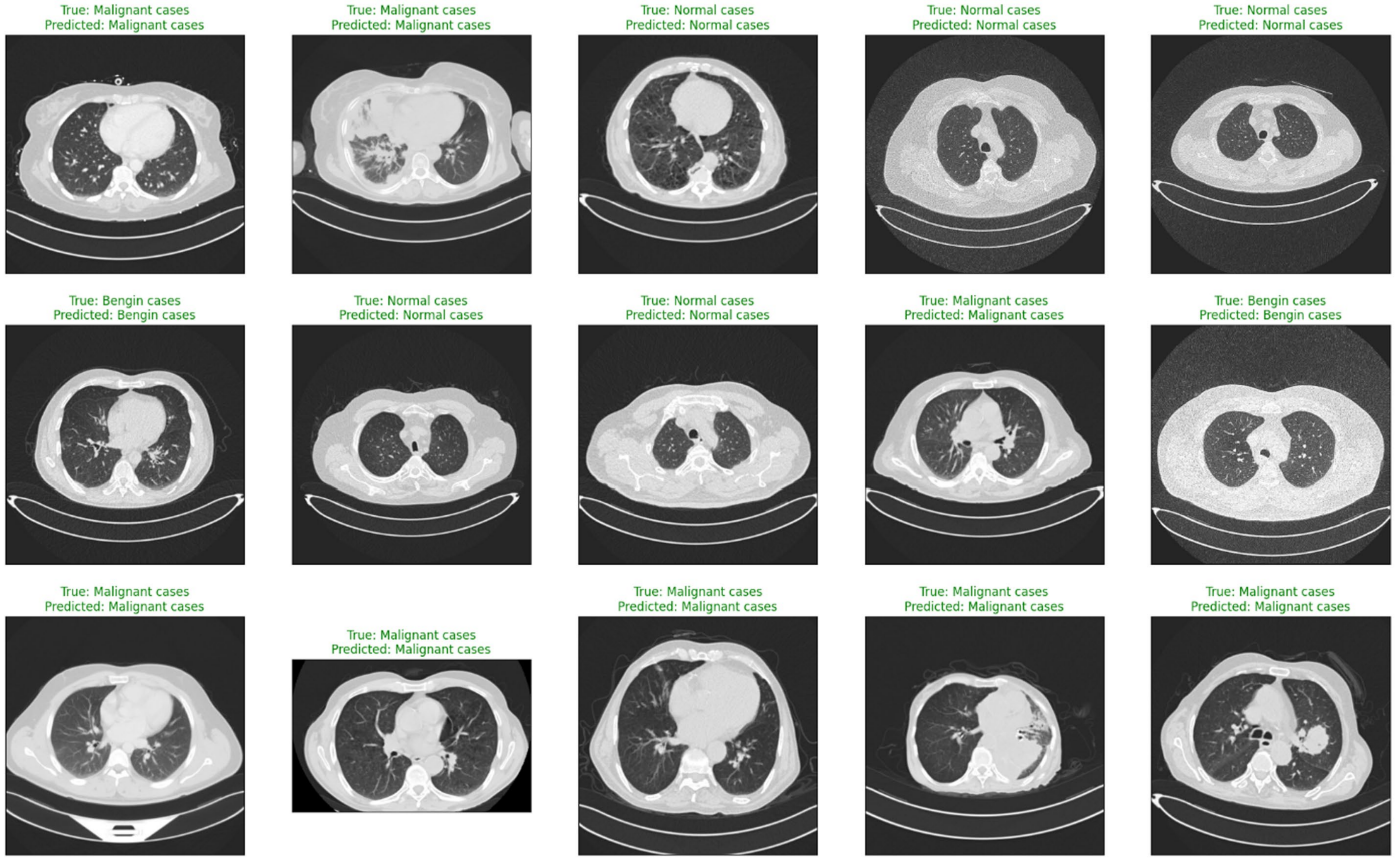
Sample CT images showing true labels versus model predictions.

When comparing the recommended AI-driven diagnostic model for lung cancer with traditional diagnostic methodologies, certain gains show, with major downsides that demand thorough examination. Conventional diagnostic methods, such radiologists manually interpreting CT images, heavily depend on the skills and knowledge of medical experts. Although these methods have long served as the foundation of medical diagnostics, their diagnostic accuracy can vary, and they frequently involve laborious procedures. With accurate predictions for most cases in several categories, Figure 8 illustrates the model’s effectiveness in detecting lung cancer stages.

**Figure 9:**
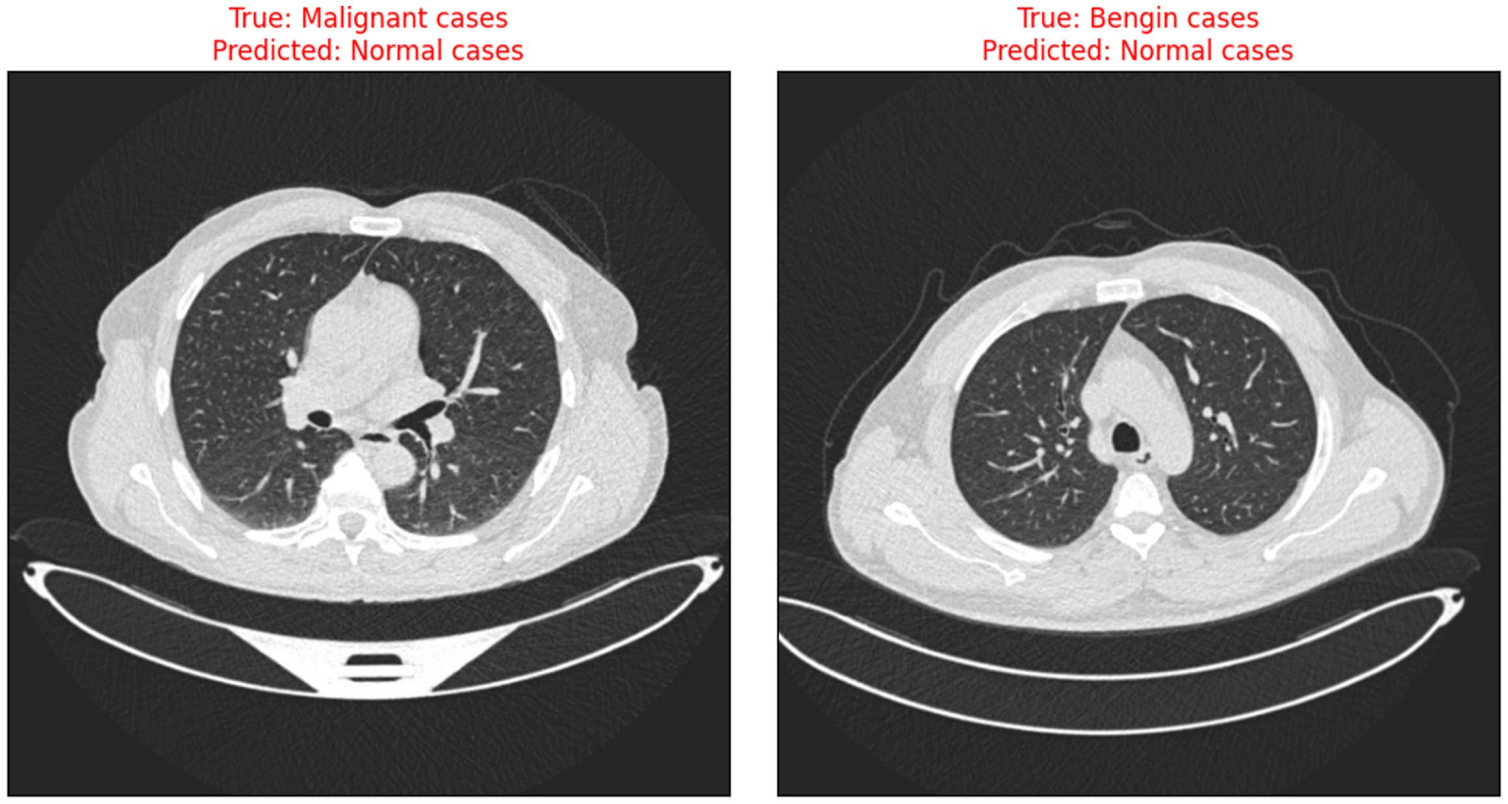
Examples of misclassified CT scans.

The risks associated with misclassification, particularly false negatives in malignant cases are significant in clinical settings. It is crucial that such tools are used as decision-support systems rather than standalone diagnostic solutions, and that their outputs are always interpreted by qualified clinicians. Transparent reporting of model performance and clear communication of its limitations are essential to minimize patient harm and uphold ethical standards in medical AI. Figure 9 illustrates the instances when the model incorrectly classified benign and malignant cases as normal, thus indicating the need for further improvement in distinguishing between minor differences in CT scan images.

The AI model is special in its use of Grad-CAM, which visually stresses the CT scan areas affecting diagnosis decisions so increasing openness. Rather than a replacement, this ability provides clinicians with perceptive examination of the AI’s decision-making process, therefore enhancing traditional diagnostic techniques. To underline this fact, Table 6 demonstrates how the proposed model surpasses the present state of art models.

**Table 6:** Comparison of the proposed model with existing models.

Study	Technique	Accuracy
Mohamed et al. (21)	Hybrid CNN with Ebola Optimization Search Algorithm (EOSA)	93.21%
Parveen et al. (22)	CNN with Watershed and SIFT for feature extraction and data augmentation	97%
Nigudgi and Bhyri (23)	Hybrid-SVM with transfer learning using AlexNet, VGG, and GoogleNet	97%
Tasnim et al. (24)	Deep Learning with advanced image preprocessing and classifiers like ResNet50 and InceptionV3	98%
Bagheri Tofighi et al. (25)	MobileNetV2 with stacked GRU layers and explainable AI using Grad-CAM	96.83%
Patnaik et al. (26)	Mask-EffNet using EfficientNet and masked autoencoder for feature extraction and classification	98.98%
Humayun et al. (27)	Transfer learning approach with CNN and various preprocessing techniques	98.83%
Bangare et al. (28)	CNN for computer-aided detection and classification of CT images	86.42%
Kumaran et al. (29)	Ensemble transfer learning using VGG16, ResNet50, and InceptionV3 with Grad-CAM	98.18%
Ahnaf and Wahyuni (30)	Comparative analysis using GLCM and LBP feature extraction with SVM and Gaussian Naive Bayes	93%
Proposed Model	Modified EfficientNet-B0 with Extra Convolution Layer and Explainable AI	99%

The achieved classification accuracy of 99% indicates substantial potential for reducing missed or incorrect lung cancer stage diagnoses. Enhanced diagnostic reliability can support timely clinical interventions, particularly in early-stage cases where therapeutic outcomes are most favorable. By decreasing human error and inter-observer variability, the model may contribute to more consistent and effective patient management.

Despite these promising results, several limitations must be addressed before clinical application (31). The potential for overfitting to a limited dataset, coupled with demographic and scanner-specific biases, constrains the model’s generalizability. The absence of external and prospective validation raises concerns regarding performance in real-world clinical settings, where variations in imaging protocols, patient populations, and unforeseen artifacts are common. Comprehensive multi-center validation and prospective clinical studies are therefore essential to establish clinical utility.

While the model demonstrates a high score of 0.98 for the normal class, even minor reductions in sensitivity or specificity could have significant clinical consequences. False positives may lead to unnecessary diagnostic procedures and patient anxiety, whereas false negatives risk delaying critical treatment. Maintaining high precision and recall across all classes is therefore imperative to minimize patient harm and resource misallocation.

Future work should prioritize robust validation strategies, including k-fold cross-validation and evaluation on independent external datasets from diverse institutions and populations. Such approaches are necessary to detect potential overfitting, enhance robustness, and more accurately estimate real-world performance. Additionally, prospective studies comparing model outputs with radiologist assessments within clinical workflows will be critical for regulatory approval and successful integration into routine practice. Despite the high performance observed, confirmation of model stability and effectiveness across larger, multi-institutional cohorts remains essential for widespread clinical adoption.

## 6 Conclusion

The model created for lung cancer classification based on CT scans shows spectacular accuracy and dependability, ratifying the enormous potential of AI-driven medical diagnosis. Grad-CAM increases model transparency and certainty and allows it to provide high-accuracy diagnoses through visualization of its decision process. Explainable AI is also a critical aspect in clinical environments as it enables medical experts to comprehend and assess AI-produced results, thereby bridging the difference between state-of-the-art AI technology and realistic clinical use. Enhanced comprehension of medical picture processing and automation in general should be the major area of research in the future. This includes broadening the scope of AI applications to cover more advanced and varied medical conditions, enhancing the resilience of AI models against varied and multi-source data, and expanding the methods for explainable artificial intelligence to improve the capture of AI findings. Due to the relatively small size of the dataset, there is still the possibility of overfitting despite the very high performance noted. For wide applicability and generalizability, It is highly recommended future validation on larger independent datasets of different centers.

## Data Availability

The original contributions presented in the study are included in the article/supplementary material, further inquiries can be directed to the corresponding authors.
