# Correction: Automated classification and explainable AI analysis of lung cancer stages using EfficientNet and gradient-weighted class activation mapping

**DOI:** 10.3389/fmed.2025.1718206

**Published:** 2025-10-15

**Authors:** Abdulmajeed Alqhatani, T. K. S. Rathish Babu, T. R. Mahesh, Surbhi Bhatia Khan, Oumaima Saidani, Mohammad Tabrez Quasim

**Affiliations:** ^1^Department of Information Systems, College of Computer Science and Information Systems, Najran University, Najran, Saudi Arabia; ^2^Department of Computer Science and Engineering, SRM Institute of Science and Technology, Ramapuram, Chennai, India; ^3^Department of Computer Science and Engineering, JAIN (Deemed-to-be University), Bengaluru, India; ^4^School of Science, Engineering and Environment, University of Salford, Salford, United Kingdom; ^5^Division of Research and Development, Lovely Professional University, Phagwara, India; ^6^Department of Information Systems, College of Computer and Information Sciences, Princess Nourah Bint Abdulrahman University, Riyadh, Saudi Arabia; ^7^Department of Computer Science and Artificial Intelligence, College of Computing and Information Technology, University of Bisha, Bisha, Saudi Arabia

**Keywords:** lung cancer staging, EfficientNet, explainable artificial intelligence, Gradient-weighted class activation mapping (Grad-CAM), CT image classification, diagnostic imaging

Author Abdulmajeed Alqhatani was erroneously spelled as Abdulmajeed Alqhatni.

In the Funding and Acknowledgements statements, an incorrect number was provided for the Growth Funding Program grant from the Deanship of Graduate Studies and Scientific Research at Najran University. The correct number is (NU/GP/SERC/13/575-5).

The original version of this article has been updated.

